# Standardized extract of *Ficus deltoidea* stimulates insulin secretion and blocks hepatic glucose production by regulating the expression of glucose-metabolic genes in streptozitocin-induced diabetic rats

**DOI:** 10.1186/1472-6882-14-220

**Published:** 2014-07-04

**Authors:** Elham Farsi, Mariam Ahmad, Sook Yee Hor, Mohamed B Khadeer Ahamed, Mun Fei Yam, Mohd Zaini Asmawi

**Affiliations:** 1 Department of Pharmacology, School of Pharmaceutical Sciences, Universiti Sains Malaysia, 11800 Penang, Malaysia; 2 Department of Physiology, School of Pharmaceutical Sciences, Universiti Sains Malaysia, 11800 Penang, Malaysia

**Keywords:** *Ficus deltoidea*, Streptozotocin, Diabetes, C-glycosylflavone, Glucose metabolism regulating genes, RT-PCR

## Abstract

**Background:**

Recently, there has been increasing interest in *Ficus deltoidea* Jack. (Moraceae) due to its chemical composition and the potential health benefits. The present study was undertaken to investigate the effect of extracts of *F. deltoidea* leaves on diabetes.

**Methods:**

The petroleum ether, chloroform and methanol extracts of *F. deltoidea* were prepared and subjected to standardization using preliminary phytochemical and HPLC analysis. Dose selection was made on the basis of acute oral toxicity study (50–5000 mg/kg b. w.) as per OECD guidelines. Diabetes mellitus was induced with streptozotocin and rats found diabetic were orally administered with the extract (250, 500 and 1000 mg/kg) for 14 days. Levels of blood glucose and insulin were measured in control as well as diabetic rats on 0, 7 and 14^th^ day. In addition, glucose metabolism regulating gene expression was assessed using RT-PCR.

**Results:**

HPLC analysis revealed that the methanol extract is enriched with C-glycosylflavones particularly, vitexin and isovitexin. In oral glucose tolerance test, oral administration of the methanol extract increased the glucose tolerance. The methanol extract showed significant (P < 0.01) antidiabetic activity. The extract treatment caused significant reduction (p < 0.01) in elevated fasting blood glucose level in streptozotocin-induced diabetic rats. The streptozotocin-related weight loss in rats was noticeably reversed by the extract treatment. Finally, RT-PCR analysis revealed a novel mechanisms for the anti-diabetic action of methanol extract of *F. deltoidea*. The extract exerted its effect via an increase of insulin secretion which impeded the hepatic glucose production, via down-regulation of phosphoenolpyruvate carboxykinase and glucose-6-phosphatase genes expression on one hand, and up-regulation of hepatic GK and PPARγ genes expression on the other hand. The extract caused an increased expression of GLUT-4 gene expression in skeletal muscles which leads to normalize the hyperglycemia. The extract also nullified the toxic effects of streptozitocin by blocking its entry into the islet β-cells through reducing the expression of GLUT-2 gene.

**Conclusion:**

It can be concluded that, *F. deltoidea* could potentially inhibits the streptozitocin-induced hyperglycemia in rats. Further the herb can be utilized as useful remedy for alleviation of diabetes complications.

## Background

Diabetes is a disease characterized by chronic hyperglycemia resulting from impaired carbohydrate metabolism with an absolute or relative lack of insulin [1]. Insulin is the key regulator of glucose uptake. The rise in insulin level in the body leads to the suppression of hepatic glycogenolysis and stimulates insulin-dependent glucose uptake into muscle, liver and adipose tissue. The rate of glucose uptake into muscle cells is dependent on the action of insulin on the glucose transporter-4 (GLUT4) [2]. The GLUT4 receptor is localized in intracellular vesicles, but when insulin binds to its own membrane receptor, GLUT4 is transported to the plasma membrane to permit glucose uptake into muscle cells.

In particular, the liver plays a vital role in maintaining glucose homeostasis and glucose-transporter-2 (GLUT2) is the main facilitative glucose transporter in the liver [3]. GLUT2 carries out insulin-independent transport of glucose. The equilibrium between extracellular and intracellular glucose concentration is maintained due to the high capacity of GLUT2 for glucose. Glucose absorption activates hepatic glucokinase (GK) which phosphorylates glucose, trapping it in liver cells for conversion to glycogen during the postprandial state. During fasting, liver produces glucose via glycogenolysis or by gluconeogenesis from non-carbohydrate precursors, such as lactate, amino acids and glycerol [3]. The rate of glycogenesis is due to the phosphorylase which is controlled by hormones whereas the rate of gluconeogenesis is controlled principally by the activities of unidirectional enzymes, such as phosphoenolpyruvate carboxykinase (PEPCK) and glucose-6-phosphatase (Glc-6-Pase). PEPCK catalyses the conversion of oxaloacetate to phosphoenol pyruvate (PEP), a rate limiting step of gluconeogenesis. Whereas, Glc-6-Pase catalyzes the final step of gluconeogenesis, the production of free glucose from glucose-6-phosphate (G6P). In addition, the expression of PEPCK and GK the key enzymes of liver gluconeogenesis and glycolysis, is under the control of insulin [4]. It is reported that insulin also regulates GLUT2 expression in the liver [5]. However, glucagon affects glucose metabolism mainly by inducing glycogen breakdown and glucose-6-phosphatase on one hand whereas, on the other hand glucagon contributes the hyperglycemia by inhibiting the enzyme pyruvate kinase [6].

Diabetes mellitus is the chief contributor to global morbidity and mortality. It is a metabolic disorder that is characterized by hyperglycemia. Among the several factors, deficiency in insulin secretion is one of the principal factors that leads to hyperglycemic condition. Emerging evidence indicates that a nuclear receptor, the peroxisome proliferator-activated receptor γ (PPARγ), enhances insulin signaling and glucose uptake in muscle upon binding PPARγ agonists [7]. Pharmacological evidence indicates that PPARγ is the major target for glitazones (TZDs). Several hypoglycemic agents that increase the insulin sensitivity, such as metformin, gliclazide and glitazones are currently being used to normalize glucose metabolism in diabetes treatment. However, the limitations related with these drugs, including adverse effects and excessive cost, have spurred the investigations of new therapeutic agents derived from plants. Due to worldwide growth of herbal products as alternative medicine, their efficacy and safety has become great concern. In recent years much attention has been paid to phytochemicals, which are widely distributed in medicinal plants and easily metabolized by humans, due to their negligible side effects and comparatively lower cost than synthetic drugs [8,9]. However, the therapeutic mechanism of these phytochemicals is not yet well clarified; therefore more attention has been drawn to evaluate the preventive and therapeutic effects of herbal medicines in the suitable experimental models.

*Ficus deltoidea* Jack. (Moraceae) is a native wild evergreen shrub in Malaysia and widely distributed in Southeast Asian countries. It is locally known as Emas Cotek and Mas Cotek [10]. Decoction of *F. deltoidea* leaves was extensively utilized in folk medicine to decrease symptom of diabetes, hyperlipidemia and hypertension [11]. Herbal healers often recommend leaves of both male and female species as libido booster, aphrodisiac, also as postpartum treatment to strengthen the uterus [12]. The beneficial effects of *F. deltoidea* on hepatoprotection, hypertension, inflammation, ulcer, wound healing, inhibition of carbohydrate hydrolyzing enzyme and antinociceptive activities were verified [10,11,13].

Although the herb is widely used in traditional medicine to treat diabetes and recent studies revealed that the herb has potential antidiabetic property [13,14], yet the possible mechanism responsible for the antidiabetic property of *F. deltoidea* is not elucidated. However, the recent studies correlated the remarkable antioxidant and α-glucosidases inhibitory activities of *F. deltoidea* with its antidiabetic property [10,15]. As circumstance this study aims to investigate the possible mechanism responsible for glucose lowering effect of *F. deltoidea* by studying the expression of GK, Glc-6-Pase, PEPCK, GLUT2 and PPARγ genes in liver and GLUT4 in muscle of streptozitocin-induced diabetic rats. To establish relation between pharmacological properties and phytochemical content of plant extract, the screening and quantitative measurement of two active principles, vitexin and isovitexin was performed.

## Methods

### Preparation of *F. deltoidea* leaf extracts

The leaves were purchased from Herbagus Sdn. Bhd. Penang-Malaysia. The floral characteristics of *F. deltoidea* were studied and confirmed by a Senior Botanist Mr. Shanmugan, School of Biological Sciences, USM. The specimens (Voucher No.: 11204) were then deposited at the herbarium of the School of Biology, Universiti Sains Malaysia. Oven dried leaves were pulverized to a fine powder and chemical components were extracted in petroleum ether, chloroform, methanol and water successively at 37°C - 40°C. All the extracts were concentrated *in vacuo* using rotavapor (Buchi Rotavapor Model R-210, Flawil, Switzerland). The extraction yield calculated was 4.7% (petroleum ether extract), 3.6% (chloroform extract), 8.2% (methanol extract) and 5.5% (water extract). All the extracts were lyophilized and kept in tightly closed glass containers and stored at -80°C until the time of use.

For *in vivo* studies, all the extracts were dissolved in 0.5% carboxymethyl cellulose (CMC) to obtain 1000 mg/mL stock solution and stored at 4°C. Further dilutions were prepared using distilled water to obtain the final concentrations of 500 and 250 mg/ml. The vehicle, 0.5% CMC is used as negative control.

### Animals

For acute toxicity study, healthy adult female Sprague–Dawley rats (200–225 g) were used. Rats (200–230 g) of both sexes were used for pharmacological studies. All the animals were maintained in polypropylene cages at room temperature for adaptation. The rats were fed with standard rodent diet *ad libitum* and had free access to tap water. The experimental procedures of the animals were approved by the Animal Ethics Committee, Universiti Sains Malaysia (AECUSM) with reference number of PPSG/07 (a)/044/(2010).

### Acute toxicity study

The study was conducted according to the OECD guideline (425) by Up-and-Down Procedure as reported earlier [16]. Separate groups of rats were orally treated with five different single doses (55, 175, 550, 1750, 5000 mg/kg) of the extract. After the oral administration of the extract, signs of possible toxicity were observed every hour for the first six hours and every day for 14 days. The body weights of all animals were recorded daily. Treatment related mortality or signs of toxicity were observed for up to 14 days. At the end of the study, LD_50_ value for the extract was estimated. For pharmacological studies three doses were selected. One was a safer dose (1/10^th^ of LD_50_ = 500 mg/kg), second was higher than the safer dose (1000 mg/kg) and third was lower than the safer dose (250 mg/kg).

### Phytochemical analysis of *F. deltoidea* leaf extracts

Phytochemical such as protein, polysaccharide, glycosaponin, phenolics, flavonoids and tanins were analyzed in the *F. deltoidea* leaf extracts. The total contents of proteins, polysaccharides and glycosaponin in the extracts were estimated calorimetrically, according to the previous study [16]. Total phenolics were determined using the Folin-Ciocalteau reagent with gallic acid as a standard and the results were expressed as mg of gallic acid equivalents, whereas total flavonoids were determined using the AlCl_3_ colorimetric method with quercetin (QNT) as a standard and the results were expressed as μg of QNT equivalent. The amount of total condensed tannins was expressed as (+) catechin equivalents [CT, mg (+) catechin/g sample].

### High performance liquid chromatography (HPLC) analysis of the extracts

A 100 mg portion of the extracts of F. deltoidea was dissolved in 15 ml methanol and subjected to ultrasonication for 10–15 min. Then the volume of the contents was made up to 25 ml using a volumetric flask. Finally, all the samples were filtered through a 0.45 mm Whatman filter paper. Similarly, the reference standards (5 mg) were dissolved in 5 ml methanol and then filtered. The stock solutions were used to prepare further dilutions.

Vitexin and isovitexin concentrations were determined by an Eclipse C18 reverse-phase (250-mm × 4.6 mm) column in an HPLC system equipped with a UV detector, quaternary pump, online degasser, auto sampler, automatic injector, column heater and photodiode array detector (Agilent Tech, Palo Alto, CA). The contents of vitexin and isovitexin were determined by the HPLC method described by Fu *et al.,*[17]. The calibration curves were set up at 330 nm by subjecting the isovitexin and vitexin standard solutions to the isocratic mobile phase, which consisted of methanol/water/formic acid (33:66.37:0.67, v/v/v), at a flow rate of 1 ml/min at 30°C with an injection volume of 10 μl. Identification and calibration of the samples were performed by comparing absorbance spectra and retention times with those of the standard reference. The concentrations of vitexin and isovitexin in the samples were estimated based on the regression lines of vitexin and isovitexin in the range of 5–200 μg/ml, which were Y = 28.305 X. 28.245 (R2 = 0.9982, n = 6) and Y = 23.90 X. 65.442 (R2 = 0.9992, n = 6), respectively, where Y is the peak area of the analyte and X is the concentration of the analyte (μg/ml).

### Assessment of hypoglycemic activity of *F. deltoidea* leaf extracts in rats

Overnight-fasted rats were divided into 6 groups of 6 rats each. Group I served as the negative control and received vehicle (0.5% of CMC) via oral gavage. Group II served as the positive control and was orally administered with glibenclamide (10 mg/kg body weight). The animals of groups III, IV, V and VI received 1000 mg/kg of petroleum ether, chloroform, methanol and water extracts, respectively via oral gavage. Fasting blood glucose level was measured initially using an ACCU-CHEKR Advantage blood glucose meter. The blood samples were collected from the tail vein at 1, 2, 3, 5 and 7 h after administering the extracts to the overnight-fasted rats [18].

### Assessment of antihyperglycemic activity of *F. deltoidea* leaf extracts in rats

The antihyperglycemic activity was assessed using an oral glucose tolerance test that was performed in the overnight-fasted rats [19]. The rats were divided into six groups of six rats each. Group I served as the negative control and received 10 ml/kg of vehicle (0.5% of CMC) orally. Group II served as the positive control and was orally administered with 500 mg/kg metformin. The animals of groups III, IV, V and VI received 1000 mg/kg of petroleum ether, chloroform, methanol and water extracts, respectively, via oral gavage daily. The Fasting blood glucose levels were checked initially using an ACCU-CHEKR Advantage blood glucose meter. The rats were then dosed intraperitoneally with 500 mg/kg of glucose one hour after administration of extracts and their blood glucose levels were examined again at 0, 30, 60, 90 and 120 min after glucose loading.

### Assessment of blood glucose and plasma insulin levels of *F. deltoidea* leaves extracts in the streptozitocin-induced diabetic rats

Diabetes was induced in 50 overnight-fasted rats with free access to water by a single IP injection of freshly prepared streptozitocin in ice-cold normal saline at a dose of 55 mg/kg. After 72 h, the rats with a blood glucose level of 15 mM were considered diabetic and used in the experiment. Diabetic rats were divided into seven groups of six rats each (n = 6). Group I consisted of normal rats that received 10 ml/kg of vehicle. Group II consisted of diabetic rats that received 10 ml/kg of vehicle. Group III of diabetic rats served as positive control and received 500 mg/kg metformin. Groups IV, V, VI and VII consisted of diabetic rats treated with 1000 mg/kg of petroleum ether, chloroform, methanol and water extracts, respectively. The treatment was given twice a day for fourteen days. Separate groups of diabetic rats were also treated with 500 mg/kg or 250 mg/kg of the most active extract (methanol extract), administered as described above. Tail vein blood samples were collected on days 1, 3, 6, 9, 12 and 15 for blood glucose analysis, and the body weights were taken [20]. The blood samples collected were also used to analyze the fasting plasma insulin levels using the Mercodia Ultra-sensitive Rat Insulin ELISA kit (Sweden). The animals from vehicle and.

### Assessment of expression of diabetic-associated gene transcripts in the streptozitocin-induced diabetic rats

To assess the effect of methanol extract of *F. deltoidea* leaves on expression of diabetic-associated gene transcripts, diabetes was induced in 20 overnight-fasted rats by a single IP injection of freshly prepared streptozitocin in ice-cold normal saline at a dose of 55 mg/kg. After 72 h, the rats with a blood glucose level of 15 mM were considered diabetic and used in the experiment. Diabetic rats were divided into 2 groups of six rats each (n = 6). Group I received 10 ml/kg of 0.5% carboxymethyl cellulose which considered as untreated group. Group II received 1000 mg/kg of methanol extract of *F. deltoidea*. The samples were orally administered twice daily for 2 weeks to diabetic rats. At the end of the experiment, all the rats were euthanized using carbon dioxide. Liver and skeletal muscle tissues were removed under aseptic conditions and immediately frozen in liquid nitrogen. One hundred milligrams of each tissue was homogenized in 1 ml TRI REAGENTR® and total RNA was extracted, following the manufacturer’s instructions (Molecular Research Center Inc, USA). Total RNA concentrations were determined by reading the absorbance at 260/280 nm using an Eppendorf spectrophotometer. The RNA integrity was verified by electrophoresis on 1% denaturing agarose gels followed by gel green staining. Single-strand cDNA was generated from total RNA (1 μg) with the RevertAid First Strand cDNA synthesis kit, following the manufacturer’s instructions (Fermentas, International Inc. Turkey). One μl of reverse transcription product was used to amplify the GK, PEPCK, Glc-6-Pase, GLUT2, GLUT4 and PPARγ genes in RT-PCR. The RT-PCR reactions were initiated at 94°C for 10 min followed by 40-cycles of denaturation at 94°C for 20 sec, primer annealing at 55°C for 20 sec, and extension at 72°C for 30 sec. β-actin was used as an internal control for the PCR. The primer sequences of the PCR are given in Table 1.

**Table 1:** List of primers, the primer sequences and the primer melting temperature (Tm)

**Gene**	**Primer probe**	**Sequence**	**Annealing Tm°C**
**GK**	Forward	5′-CATATGTGCTCCGCAGGACTAG-3′	61.7
	Reverse	5′-CTTGTACACGGAGCCATCCA-3′	
**PEPCK**	Forward	5′-GCAACTTCTCTCGGCTCGTT-3′	62.7
	Reverse	5′-TGGCAGTTCTACTGGGCTACAC-3′	
**G6Pase**	Forward	5′-GGATCTACCTTGCGGCTCACT-3′	62.7
	Reverse	5′-TGTAGATGCCCCGGATGTG-3′	
**GLUT 2**	Forward	5′-CATCAAAACGTAGAGCACGGTAA-3′	63.4
	Reverse	5′-TATGGGCATTTAGTCTGCACGTA-3′	
**GLUT4**	Forward	5′-GCTTGGCTCCCTTCAGTTTG-3′	63.4
	Reverse	5′-CCTACCCAGCCAAGTTGCAT-3′	
**PPAR γ**	Forward	5′-CATGAGTTCTTGCGCAGTATCC-3′	63.4
	Reverse	5′-AGAGCATTGAACTTGACAGCAAAC-3′	
**Β-actin**	Forward	5′-GCTCTGGCTCCTAGCACCAT-3′	55
	Reverse	5′-GCTGATCCACATCTGCTGGAA-3′	

GK: Glucokinase, PEPCK: Phosphoenolpyruvate carboxykinase, G6Pase: Glucose-6-phosphatase, GLUT2: Glucose transporter type 2, GLUT4: Glucose transporter type 4, PPARγ peroxisome proliferator-activated receptors-gamma.

### Statistical analyses

The data are represented as the mean ± SEM. The differences between treated and control groups were analyzed by one-way ANOVA followed by post hoc Dunnett’s test and accepted as significant at *p* < 0.05- 0 < 0.01.

## Results

### The total contents of proteins, polysaccharides, glycosaponins, phenolics, flavonoids and tannins in *F. deltoidea* leaf extracts

The results of quantitative analysis of total proteins, polysaccharides, glycosaponins, phenolics, flavonoids and tannins present in the extracts of *F. deltoidea* leaves is given in Table 2. The methanol extract of *F. deltoidea* showed the highest contents of phenolics, tannins, flavonoids, proteins, glycosaponins and polysaccharides. The water extract was found to be moderately rich in these phytochemicals, whereas the petroleum ether and chloroform extracts of the plant showed very few phenolics, flavonoids, tannins, polysaccharides, glycosaponins and proteins.

**Table 2:** **Phytochemical contents of *F. deltoidea *** **leaf extracts**

**Phytochemicals**	**FPE**^**a**^	**FCE**^**b**^	**FME**^**c**^	**FWE**^**d**^
**Total Phenolics (mg/g)**	0.04 ± 0.01	0.31 ± 0.02	213.10 ± 0.01	70.40 ± 0.03
**Total Flavonoids (mg/g)**	0.003 ± 0.01	0.20 ± 0.03	98.60 ± 0.12	10.10 ± 0.06
**Total Tannins (mg/g)**	ND^e^	0.30 ± 0.01	320.40 ± 9.20	86.70 ± 3.21
**Total Polysaccharides (%)**	1.8 ± 0.02	3.30 ± 0.03	6.60 ± 0.04	5.50 ± 0.05
**Total Glycosaponins (%)**	ND^e^	ND^e^	34.80 ± 1.20	7.40 ± 0.04
**Total Proteins (%)**	8.50 ± 0.01	13.90 ± 0.09	37.60 ± 2.10	32.60 ± 1.10

^a^petroleum ether extract (FPE), ^b^chloroform extract (FCE), ^c^methanol extract (FME) and ^d^water extract (FWE).

^e^ND: Not detected.

### HPLC analysis of *F. deltoidea* leaf extracts

**Figure 1:**
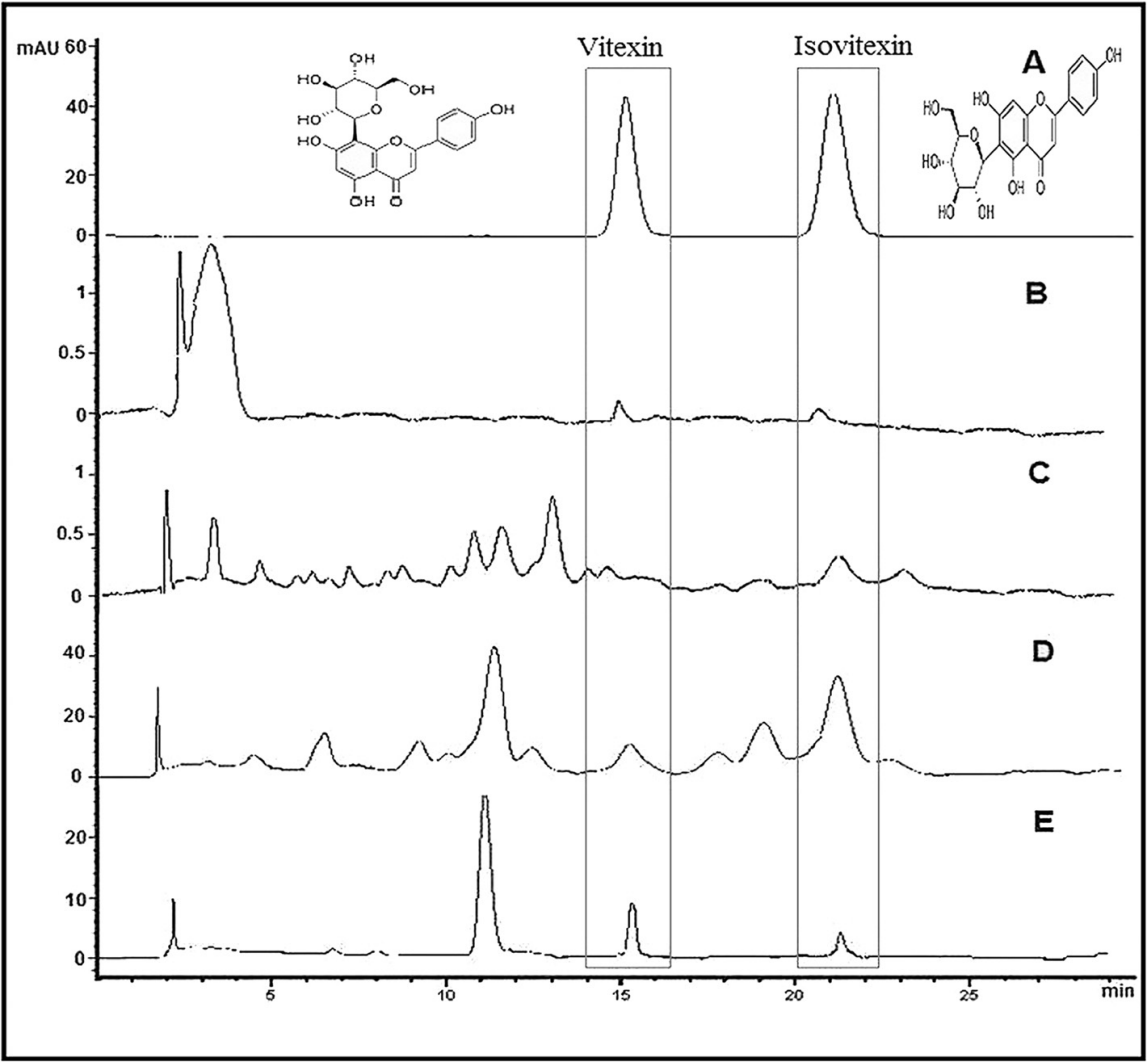
**HPLC chromatogram of vitexin and isovitexin in different leaves extracts of *F. deltoidea*. A**: vitexin and isovitexin, **B**: vitexin and isovitexin in petroleum ether extract, **C**: vitexin and isovitexin in chloroform extract, **D**: vitexin and isovitexin in methanol extract and **E**: vitexin and isovitexin in aqueous extract.

Vitexin and isovitexin in the *F. deltoidea* leaves extracts were detected and quantified by HPLC. The contents of vitexin and isovitexin varied between the different extracts of the plant (Table 3). HPLC chromatograms of the extracts taken at 330 nm along with the mixed standards are shown in Figure 1 the results indicated that the methoanolic extract showed higher contents of vitexin and isovitexin than compared to the other extracts.

**Table 3:** **Quantitative estimation of vitexin and isovitexin (mg/g) in different extracts of *F. deltoidea *** **leaf**

**Markers**	**FPE**^**a**^	**FCE**^**b**^	**FME**^**c**^	**FWE**^**d**^
Vitexin	0.69 ± 0.12	0.72 ± 0.08	6.29 ± 0.18	2.43 ± 0.02
Isovitexin	0.27 ± 0.09	0.41 ± 0.02	14.34 ± 0.26	0.98 ± 0.09

^a^petroleum ether extract (FPE), ^b^chloroform extract (FCE), ^c^methanol extract (FME) and ^d^water extract (FWE).

### Acute toxicity studies

Rats were orally treated with five different single doses of the extract and screened for signs of toxicity two weeks after administration. No treatment-related mortality was observed at all the tested doses. Throughout the 14 days observation period, no significant changes in behavior such as apathy, hyperactivity, morbidity, etc., were recorded in the treated animals. No abnormal changes attributable to the treatment were noticed in body weights, respiration rate and heart rate. The present study demonstrated that the methanol extract of *F. deltoidea* found to be safe at dose level of 5000 mg/kg and therefore, the LD_50_ value for oral toxicity is considered to be more than 5000 mg/kg.

### *F. deltoidea* leaf extracts failed to cause hypoglycemia in fasted rats but reduced hyperglycemia in glucose loaded rats

**Figure 2:**
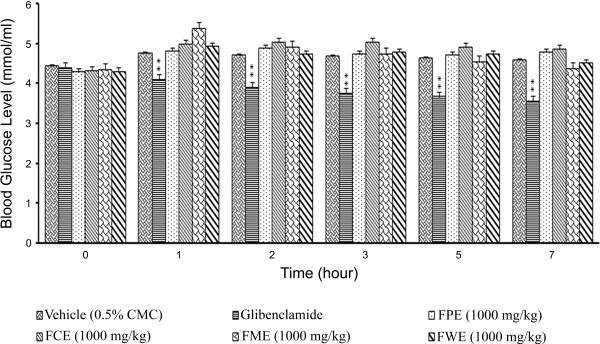
**The effect of oral administration of *F. deltoidea *leaf extracts (1000 mg/kg) on the blood glucose levels of rats dosed with 1000 mg/kg of glucose.** FPE = Petroleum ether extract; FCE = Chloroform extract; FME = Methanol extract and FEW = Water extract. The values are expressed as mean ± SEM (n = 6 in each group). **p < 0.01 compared to the control group (10 ml/kg vehicle).

Treatment of the animals with 1000 mg/kg of plant extracts did not produce any significant hypoglycemic effects (data not shown). Thus, the extracts will not cause blood glucose levels to fall below the standards required by the body. In oral glucose tolerance test, all tested plant extracts at a dose of 1000 mg/kg showed significant (*p* < 0.01) reduction in plasma glucose levels (Figure 2). Particularly, the methanol extract significantly (*p* < 0.01) reduced the plasma glucose level in a time-dependent manner. About 22.75% reduction of plasma glucose level was observed, as early as 15 min after glucose administration, whereas the plasma glucose level was further reduced by up to 33.30% after 120 min of glucose administration.

### Effect of *F. deltoidea* leaf extracts on blood glucose in streptozitocin -induced diabetic rats

**Figure 3:**
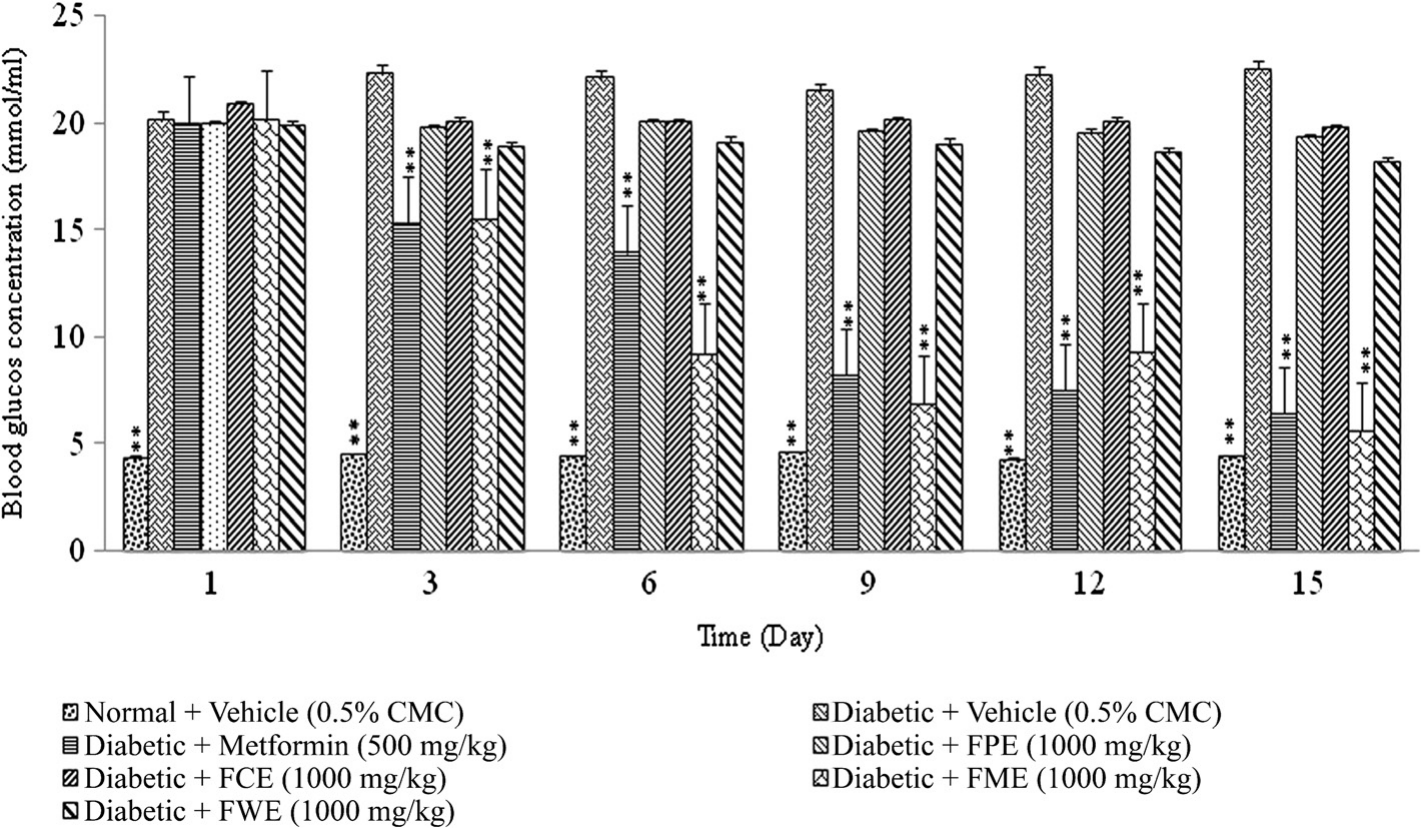
**The effect of daily oral administration of *F. deltoidea *leaf extracts (1000 mg/kg) on the blood glucose levels of streptozitocin-induced diabetic rats.** FPE = Petroleum ether extract; FCE = Chloroform extract; FME = Methanol extract and FEW = Water extract. The values are expressed as mean ± SEM (n = 6 in each group). **p < 0.01 compared to the diabetic control group (10 ml/kg vehicle).

**Figure 4:**
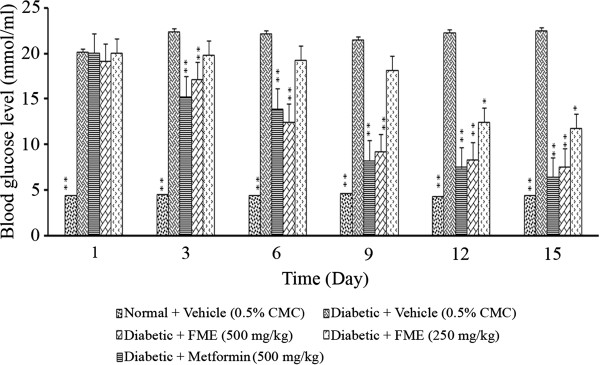
**The effect of daily oral administration of *F. deltoidea *leaf methanol extract (FME) on the blood glucose levels of streptozitocin-induced diabetic rats.** The methanol extract (FME) was administered to the animals at different doses (500 mg/kg or 250 mg/kg). The values are expressed as mean ± SEM (n = 6 in each group). *p < 0.05 and **p < 0.01 compared to the diabetic control group (10 ml/kg vehicle).

A preliminary screening test was carried out with the all the extracts at 1000 mg/kg. The result revealed that only methanol extract demonstrated significant (p < 0.01) anti-diabetic activity when compared to the negative control as well as the other test groups. The results showed that, the extract produced a marked reduction in fasting plasma glucose level in streptozitocin-induced diabetic rats. About 30.55% reduction was noticed after 3 days of oral administration of methanol extract (1000 mg/kg), whereas it was further reduced to 60.19% by the end of the experiment (after 15 days treatment). The activity was significantly (p < 0.01) comparable to that of the standard reference, metformin 71.34%. Figure 3 shows the blood glucose levels in streptozitocin-induced diabetic rats over 15 days after the administration of different extracts of *F. deltoidea* leaves.Furthermore, treatment with methanol extract at 250 and 500 mg/kg resulted in a dose-dependent depletion in blood glucose level by 13.89 and 23.14%, respectively after 3 days of repeated administration of the extract in streptozitocin-induced diabetic rats (Figure 4). The reduction in blood glucose levels was persisted throughout the period of treatment and after 15 days of treatment with methanol extract (250 and 500 mg/kg), the blood glucose level was further reduced by 41.38 and 62.63%, respectively.

### Stimulatory effect of methanol extract of *F. deltoidea* leaves on plasma insulin levels

**Figure 5:**
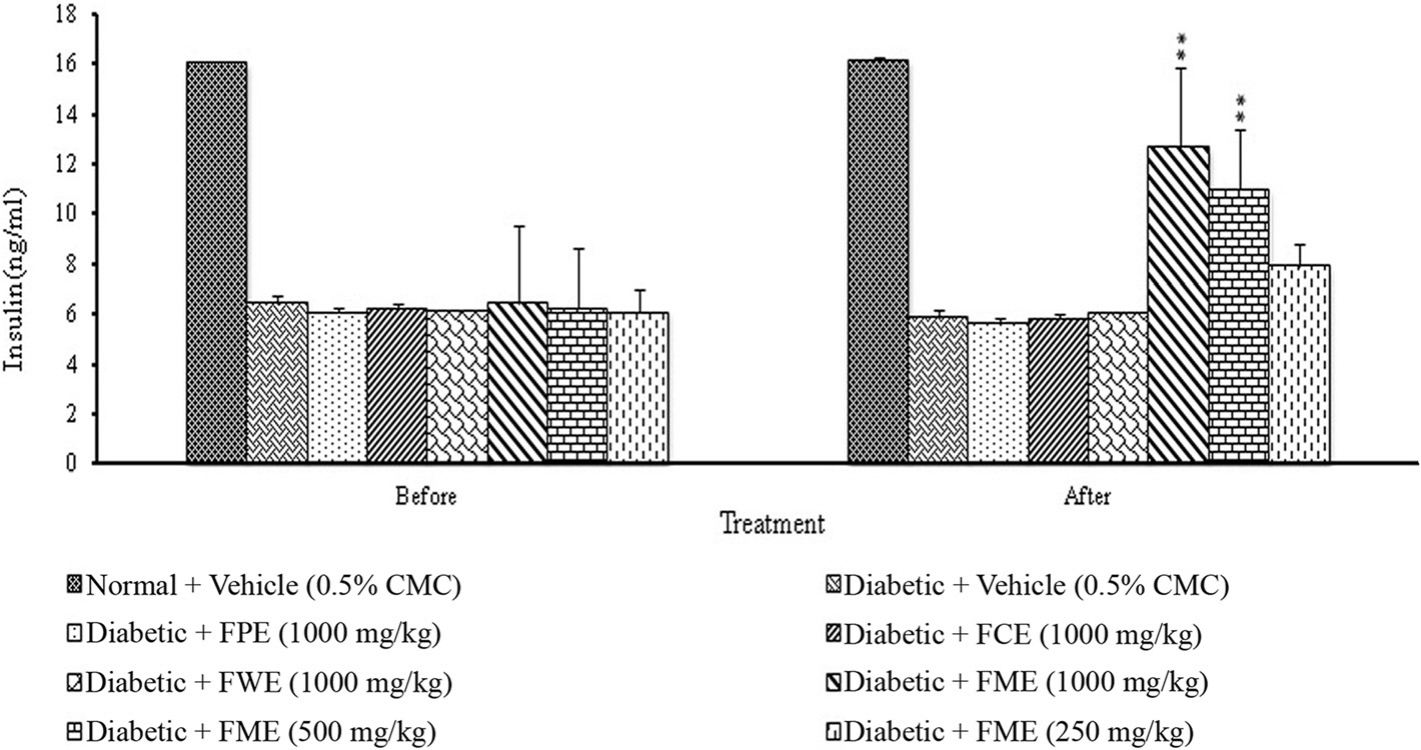
**The effect of daily oral administration of *F. deltoidea *leaf extracts on the plasma insulin level of streptozitocin-induced diabetic rats.** The data was collected on the first and the last days of extract administration. FPE = Petroleum ether extract; FCE = Chloroform extract; FME = Methanol extract and FEW = Water extract. The values are expressed as mean ± SEM (n = 6 in each group). **p < 0.01 compared to the diabetic control group (10 ml/kg vehicle).

The initial and final levels of plasma circulating insulin are presented in Figure 5. Except the control, no effect was observed in the plasma insulin levels of the animals on the first day of treatment. The plasma insulin levels in streptozitocin-induced diabetic rats were significantly increased by 34.13, 86.01 and 115% in response to the treatment with methanol extract at concentrations of 250, 500 and 1000 mg/kg, respectively.

### Effect of methanol extract of *F. deltoidea* leaves on body weights

**Figure 6:**
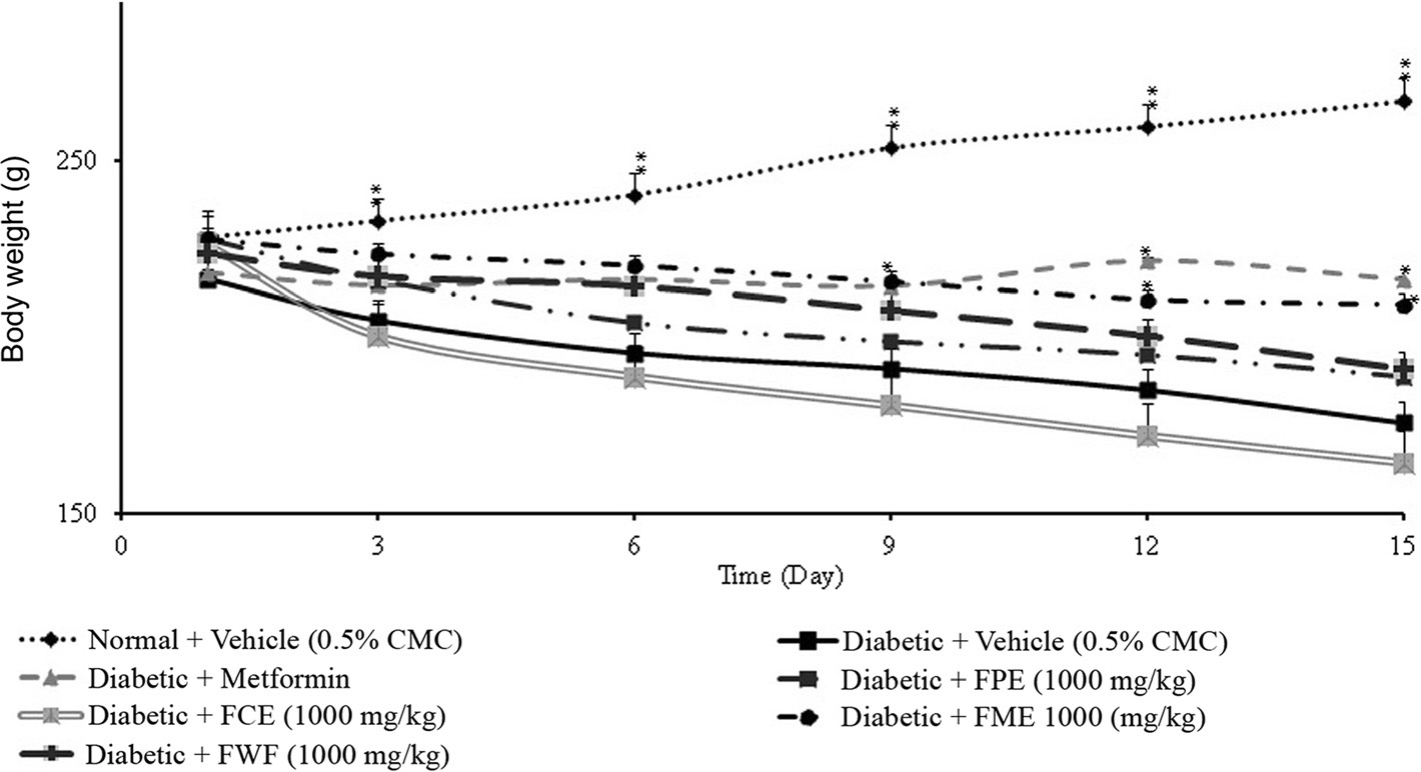
**The effect of daily oral administration of *F. deltoidea *leaf extracts (1000 mg/kg) on the body weight gain of streptozitocin-induced diabetic rats.** FPE = Petroleum ether extract; FCE = Chloroform extract; FME = Methanol extract and FEW = Water extract. The values are expressed as mean ± SEM (n = 6 in each group). *p < 0.05 and **p < 0.01 compared to the diabetic control group (10 ml/kg vehicle).

**Figure 7:**
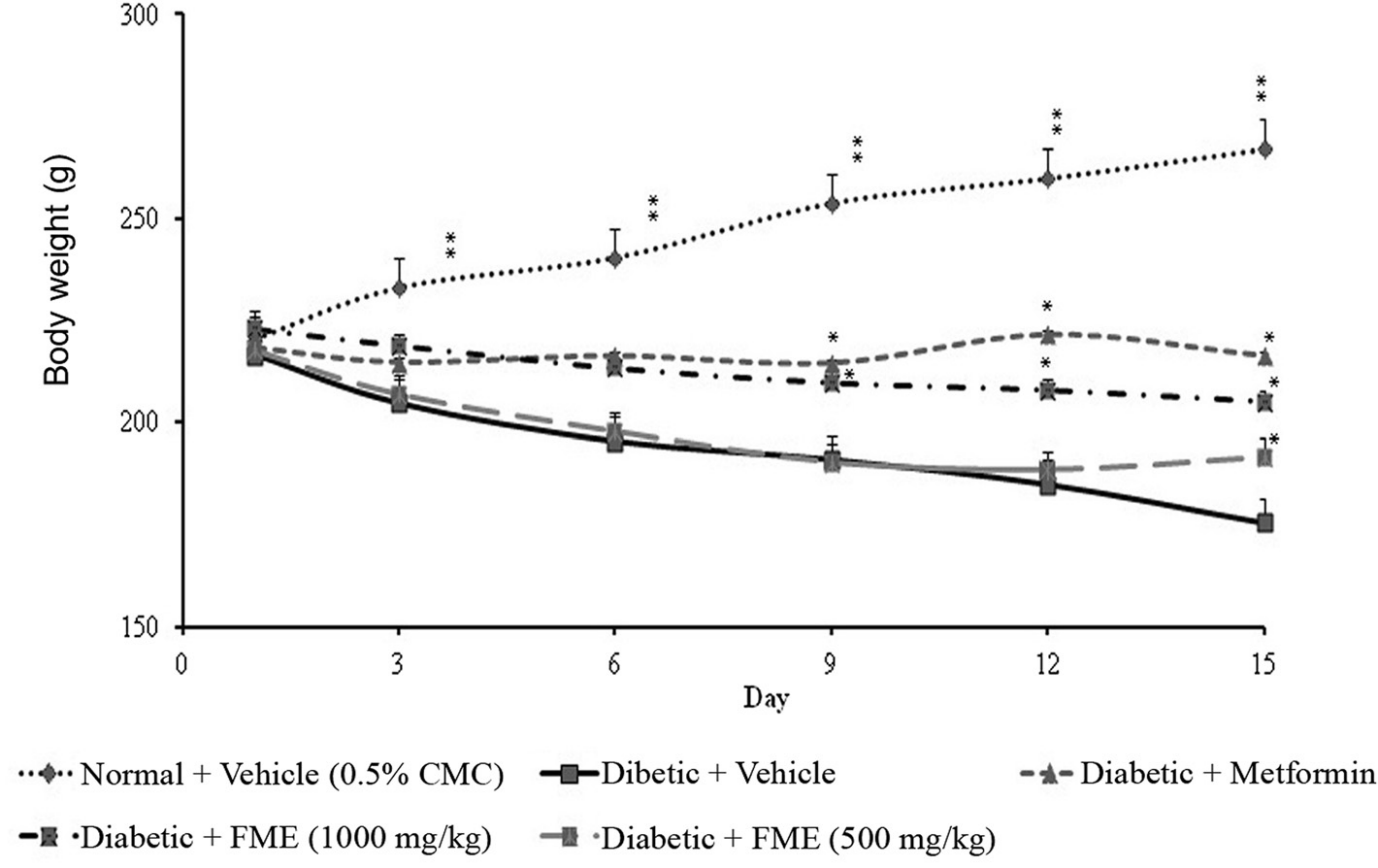
**The effect of daily oral treatment with different doses (500 mg/kg or 250 mg/kg) of *F. deltoidea *leaf methanol extract (FME) on the body weight of streptozitocin-diabetic rats.** The values are expressed as the mean SEM (n = 6 in each group). *p < 0.05 and **p < 0.01 compared to diabetic control group (10 ml/kg vehicle).

Figures 6 and 7 depicted the changes in the body weights of streptozitocin-induced diabetic rats administered with different doses of methanol extract (250, 500 and 1000 mg/kg). A significant effect of suppression in body weight gain was recorded after 9 days of treatment with 500 and 1000 mg/kg. However, the low dose (250 mg/kg) group of animals produced a significant weight loss only at the end of the experiment.

### The effect of methanol extract on the expression of diabetic-associated gene transcripts in streptozitocin-induced diabetic rats

**Figure 8:**
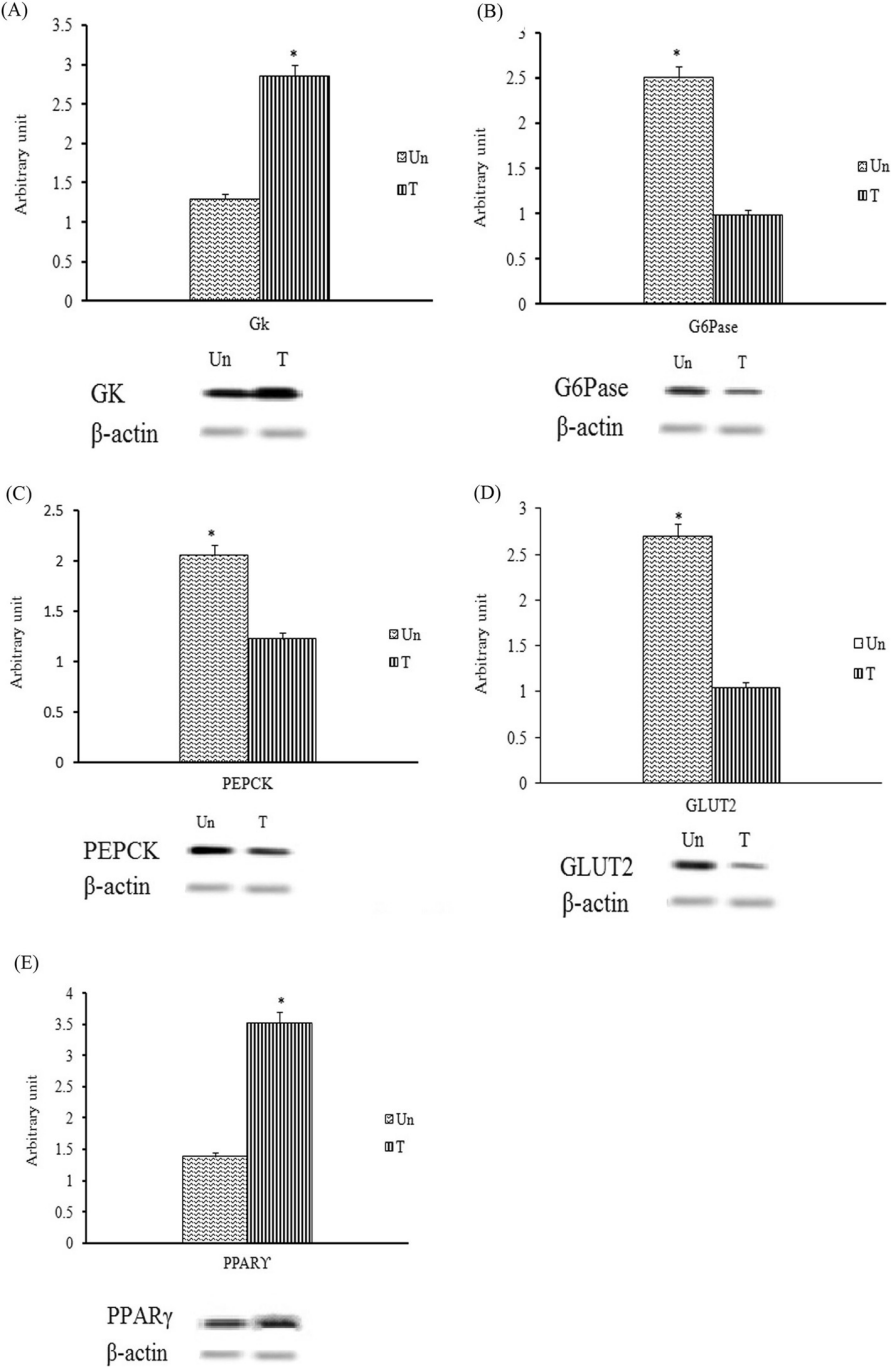
**Semi-quantitative PCR analysis of (A) GK, (B) Glc-6-Pase, (C) PEPCK, (D) GLUT2 and (E) PPARγ transcripts in the liver of untreated (Un) and methanol extract-treated (T) streptozitocin-induced diabetic rats.** PCR product bands were measured using scanning densitometry. The densities are shown as mean ± SEM from three independent experiments. *p < 0.05 was regarded as statistically significant.

**Figure 9:**
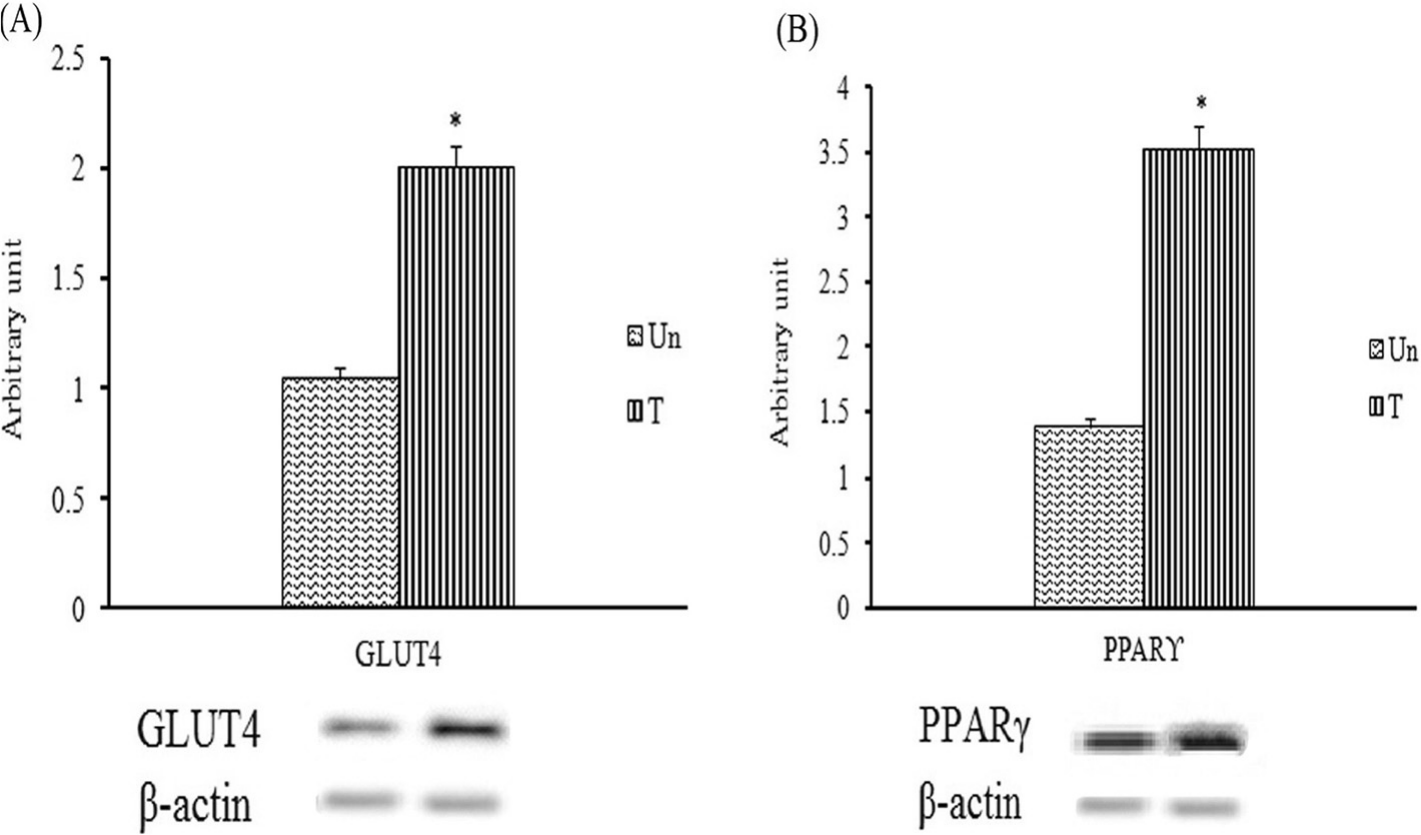
**Semi-quantitative PCR analysis of (A) GLUT4 and (B) PPARγ transcripts in the muscle of untreated (Un) and methanol extract-treated (T) streptozitocin-induced diabetic rats.** PCR product bands were measured using scanning densitometry. The densities are shown as mean ± SEM from three independent experiments. *p < 0.05 was regarded as statistically significant.

The expression of diabetic-associated, glycolytic and gluconeogenic genes, in the streptozitocin-induced diabetic rats treated with methanol extract was determined using RT-PCR. As the loading control, the band density of each target gene was normalized to the band density of β-actin of the sample. Treatment with the methanol extract significantly (*p* < 0.05) enhanced the expression of hepatic GK and PPARγ transcripts by 1.79 and 2.04-fold, respectively (Figure 8). Conversely, the treatment significantly (*p* < 0.05) reduced the expression of hepatic Glc-6-Pase, PEPCK and GLUT2 transcripts by 2.38-fold, 1.58-fold and 2.38-fold, respectively, compared to the untreated group. On the other hand, the expression of muscle GLUT4 and PPARγ showed altered expression profiles in the streptozitocin-induced diabetic rats following 2-weeks treatment of methanol extract. The expression of muscle GLUT4 transcript was significantly (*p* < 0.05) elevated by 1.9 fold compared to that of untreated group (Figure 9).

## Discussions

Regardless of the popularity of the traditional medicinal uses of *F. deltoidea* for diabetes related symptoms, very few studies have been reported to determine the molecular mechanism and genes expression involve in the remedial effects of the herb. The reports on traditional uses of the herb to treat diabetes and the evidences that the plant is naturally gifted with abundance of antioxidant-rich bioactive constituents, have drawn attention toward hypothesizing that *F. deltoidea* could be useful as a promising remedy against diabetes if its efficacy is investigated and scientifically proven using the animal models with standard experimental protocols. Accordingly, the present study was designed to evaluate the acute and chronic anti-hyperglycemic effects of *F. deltoidea* extracts in rats. The present study demonstrates that the methanol extract noticeably reduced the plasma glucose level in a time-dependent manner in oral glucose tolerance test conducted in rats. Furthermore, the methanol extract significantly decreased the fasting plasma glucose level and increased the plasma insulin level in the streptozitocin-induced diabetic rats. In addition, the extract suppressed the body weight gain in the tested animals.

Repeated (14 days) administration of the methanol extract alleviated hyperglycemia and simultaneously increased plasma insulin levels in the streptozitocin-induced diabetic rats compared with those of the control diabetic rat. The antihyperglycemic activity of the methanol extract may partly due to its anti-oxidant and antagonistic effects against the toxic effects induced by streptozitocin [21,22]. The phytochemical analysis in the present study revealed that the methanol extract of *F. deltoidea* contains the highest content of flavonoids, phenolics, and tannins. HPLC analysis showed that the extract was particularly enriched with bioactive flavone C-glycosides, vitexin and isovitexin. The presences of flavonoids, which are efficient antioxidants, play a crucial role in cytoprotection and scavenging of free radicals, thereby protect the β-cells from oxidative damage. Evidence of the C-glycosyl flavones in plants with antidiabetic properties and their efficacy in the treatment and prevention of diabetes has been extensively documented [21-24]. According to the Malaysian herbal monograph, vitexin and isovitexin are considered as the characteristic chemical markers of *F. deltoidea* leaves. In addition, it is reported that vitexin and isovitexin significantly reduced postprandial blood glucose level in mice and showed *in vivoα*-glucosidase inhibitory activity. Therefore, in the present study the activity of the extracts was correlated with quantitative estimation of the marker compounds. HPLC analysis revealed that quantity of both marker compounds was higher in methanol extract than compared to the other extracts of *F. deltoidea* leaves [25].

The antagonistic effects demonstrated by the methanol extract of *F. deltoidea* against streptozitocin-induced hyperglycemia were quite comparable with that of standard reference, metformin. Streptozitocin is a cytotoxic glucose analogue that is selectively absorbed by insulin producing β-cells through GLUT2 and it induces the death of β-cells by donating nitric oxide, generating reactive oxygen species and disrupting the actions of pancreatic antioxidant enzymes [26]. Pancreatic β-cells, which store and release insulin, are the main regulator of glycogenesis in muscle and liver. The absence of active β-cells is associated with a decrease in insulin level and an increase in peripheral insulin resistance, which results in excess glucose in the circulating blood. IP injection of streptozitocin (55 mg/kg) in this study effectively induced diabetes in normal rats. Streptozitocin injection produced a significant increase in the blood glucose level and body weight and lowered the plasma insulin level in the rats after 72 hours. On the one hand, the methanol extract was observed to reduced hyperglycemia by suppressing hepatic gluconeogenesis through inducing the expression of PPARγ and GK, and on the other hand, the extract demonstrated remarkable decreased in expression of the hepatic gluconeogenic genes PEPCK and Glc-6-Pase. These results have a similar effect as metformin in the streptozitocin-induced diabetic rats [27]. Thus it is predicted that the blood-glucose lowering effect of the methanol extract of *F. deltoidea* could be attributed to the metformin-similar mechanism of suppression of hepatic glucose output [7,27,28]. Similar to metformin, the extract improved cellular sensitivity towards insulin by activating the insulin transduction pathway and consequently, the regulation of glucose absorption and metabolism in peripheral tissues [29,30]. Similarly, repeated treatment with the methanol extract of *F. deltoidea* in streptozitocin-induced diabetic rats increased the mRNA transcript level of GLUT4 in muscles. Additionally, reduction of mRNA transcripts of hepatic Glc-6-Pase and PEPCK was also noticed in the same group of streptozitocin-induced diabetic rats. Interestingly, the expression of the glucose transporter Glut2 in liver which is insulin-independent was found to be reduced in the extract treated streptozitocin-induced diabetic rats. The decrease in GLUT2 expression is related to a decrease in hepatic glucose output and plays a critical role in glucose homeostasis [31]. GLUT2 is the sole transporter that specifically transports streptozotocin in pancrease [32]. Thus it could be postulated that methanol extract of *F. deltoidea* besides being strong antioxidant, obstructs the cytotoxic effect of streptozitocin on islet β-cells of pancreas by inhibiting GLUT2 gene expression.

Although several preliminary studies on antidiabetic effects of *F. deltoiea* have been carried out, the mechanism of action at genetic level have yet to be investigated. PPARγ has been reported to regulate the expression of genes involved in glucose metabolism [33]. In the present study, it was observed that the methanol extract enhanced PPARγ expression in the liver of diabetic rats. In turn, PPARγ can directly activate hepatic GK expression. GK works as the glucose sensor in glucose homeostasis by phosphorylating glucose to glucose-6-phosphate, is the first step for both glycogen synthesis and glycolysis [34]. In the present study, administration of the *F. deltoidea* methanol extract increased the expression of hepatic GK and therefore, it can improve excess glucose tolerance. There is a report that, over expression of hepatic GK reduces blood glucose in the body [35]. Similarly, a research study showed that the activity of Glc-6-Pase was increased in streptozitocin-induced diabetic rats [36]. In this study, Glc-6-Pase activity was markedly increased in the streptozitocin-induced diabetic rats when compared to that of normal control. However, the transcript level of Glc-6-Pase was significantly decreased after administration of the methanol extract, which probably due to the reduced extent of gluconeogenesis. This clearly indicates that methanol extract of *F. deltoidea* produces anti-hyperglycemia in the animal model by promoting glucose utilization and suppressing the glucose production [37].

## Conclusions

In conclusion, our results provide novel mechanisms for the plasma glucose-lowering action of methanol extract of *F. deltoidea*. The extract produced its anti-hyperglycemic effect via increased secretion of insulin. Further it is confirmed that the extract suppressed the transcription of genes involved in hepatic glucose production, such as phosphoenolpyruvate carboxykinase (PEPCK) and Glc-6-Pase. In addition, the extract stimulated the hepatic GK and PPARγ gene expression, which consequently leads to an increased expression of GLUT-4 gene expression in skeletal muscles of streptozitocin-induced diabetic rats. Altogether, the extract potentially displayed antidiabetic activity by inhibiting hepatic glucose production and promoting glucose utilization. The extract also nullifies the hyperglycemic effects of streptozitocin which was observed through the reduced expression of GLUT-2 gene. Altogether, it can be concluded that the methanol extract of *F. deltoidea* has the potential to be developed as an antidiabetic agent.

## Competing interests

The authors declare no financial or commercial conflicts of interest.

## Authors’ contributions

MZA, MBKA, and EF designed the experiments. EF and SYH carried out the phytochemical analysis. EF and SYH performed the *in vivo* studies. EF, MBKA, MZA and MYF participated in gene analysis and drafted the manuscript. EF, BKA and MA analyzed all data and interpreted the results. All authors read and approved the final manuscript.

## Pre-publication history

The pre-publication history for this paper can be accessed here:

http://www.biomedcentral.com/1472-6882/14/220/prepub
