# Correction to: Standardized extract of *Ficus deltoidea* stimulates insulin secretion and blocks hepatic glucose production by regulating the expression of glucose-metabolic genes in streptozitocin-induced diabetic rats

**DOI:** 10.1186/s12906-018-2333-3

**Published:** 2018-09-27

**Authors:** Elham Farsi, Mariam Ahmad, Sook Yee Hor, Mohamed B Khadeer Ahamed, Mun Fei Yam, Boon Yin Khoo, Mohd Zaini Asmawi

**Affiliations:** 1 Department of Pharmacology, School of Pharmaceutical Sciences, Universiti Sains Malaysia, Penang, Malaysia; 2 Department of Physiology, School of Pharmaceutical Sciences, Universiti Sains Malaysia, Penang, Malaysia; 3 Institute for Research in Molecular Medicine (INFORMM), Universiti Sains Malaysia, 11800 Penang, Malaysia

## Correction to: BMC Complementary and Alternative Medicine 2014, 14:220 DOI 10.1186/1472-6882-14-220

After the publication of this article [1] it came to our attention that one author, Boon Yin Khoo, was erroneously omitted from the authorship list.

The authorship list previously read:

“Elham Farsi, Mariam Ahmad, Sook Yee Hor, Mohamed B Khadeer Ahamed, Mun Fei Yam and Mohd Zaini Asmawi.”

The authorship list now reads:

“Elham Farsi, Mariam Ahmad, Sook Yee Hor, Mohamed B Khadeer Ahamed, Mun Fei Yam, **Boon Yin Khoo** and Mohd Zaini Asmawi.”

The affiliation for **Boon Yin Khoo** is as follows:

“3. Institute for Research in Molecular Medicine (INFORMM), Universiti Sains Malaysia, 11800 Penang, Malaysia.”

The Authors’ Contributions section previously read:

“MZA, MBKA, and EF designed the experiments. EF and SYH carried out the phytochemical analysis. EF and SYH performed the *in vivo* studies. EF, MBKA, MZA and MYF participated in gene analysis and drafted the manuscript. EF, BKA and MA analyzed all data and interpreted the results. All authors read and approved the final manuscript.”

The Authors’ Contributions section now reads:

“MZA, **BYK**, MBKA, and EF designed the experiments. EF and SYH carried out the phytochemical analysis. EF and SYH performed the in vivo studies. MZA, **BYK**, MBKA and EF participated in gene analysis and drafted the manuscript. BKA, EF and MFY analyzed all data and interpreted the results. All authors read and approved the final manuscript.”

It also came to our attention that the affiliation for **Elham Farsi** was incorrect.

The affiliation for **Elham Farsi** previously read:

“1. Department of Pharmacology, School of Pharmaceutical Sciences, Universiti Sains Malaysia, Penang, Malaysia.”

The affiliation for **Elham Farsi** now reads:

“1. Department of Pharmacology, School of Pharmaceutical Sciences, Universiti Sains Malaysia, Penang, Malaysia.”

“3. Institute for Research in Molecular Medicine (INFORMM), Universiti Sains Malaysia, 11800 Penang, Malaysia.”
